# Surface motion dynamics and swimming control of planar magnetic microswimmers

**DOI:** 10.1038/s41598-025-94078-y

**Published:** 2025-03-20

**Authors:** Yasin Cagatay Duygu, Sangwon Lee, Austin Liu, U. Kei Cheang, Min Jun Kim

**Affiliations:** 1 Department of Mechanical Engineering, Southern Methodist University, Dallas, TX USA; 2 The Harker School, San Jose, CA USA; 3 Department of Mechanical and Energy Engineering, Southern University of Science and Technology (SUSTech), Shenzhen, China

**Keywords:** Planar magnetic microswimmers, Surface motion dynamics, Microrobotics, Magnetic field control, Targeted drug delivery, Engineering, Biomedical engineering, Mechanical engineering

## Abstract

Planar magnetic microswimmers offer substantial potential for in vivo biomedical applications, owing to their efficient mass production via photolithography. In this study, we demonstrate the effective control of these microswimmers using an open-loop approach in environments with minimal external disturbances. We investigate their surface motion characteristics through both theoretical modeling and experimental testing under varying magnetic field strengths and rotation frequencies, identifying regions of stable and unstable motion. Additionally, we analyze how field frequency and strength influence surface motion speed and identify the frequencies that promote stability. Open-loop control of surface motion in fluid environments and swimming in channels is also demonstrated, highlighting the operational flexibility of these microswimmers. We further demonstrate swarm motion for both swimming and surface operations, exhibiting larger-scale coordination. Our findings emphasize their potential for future applications in biomedical engineering and microrobotics, marking a step forward in the development of microscale robotic systems.

**Supplementary Information:**

The online version contains supplementary material available at 10.1038/s41598-025-94078-y.

## Introduction

Recent advances in magnetic microswimmers have highlighted the importance of controlled navigation in complex environments, particularly for biomedical applications^1–3^. The increasing focus on intelligent materials, advanced actuation techniques, and control systems has driven a surge in research, with annual publications in microrobotics and nanorobotics projected to exceed 3000 and 2500, respectively^4^. Planar magnetic microswimmers, in particular, stand out due to their scalable and efficient production using standard photolithography techniques^5–7^. These microscale robotic systems hold great potential for biomedical applications like targeted drug delivery, precision surgery, and infarction of tumor blood vessels^8,9^.

Earlier research primarily concentrated on fluidic propulsion in low Reynolds number environments, drawing inspiration from microorganism movement^10–12^. While various geometries, such as helical shapes, have been investigated for propulsion efficiency, their production remains complex and often costly^13–17^. In contrast, planar magnetic microswimmers not only offer a simpler production process but also demonstrate significant potential across diverse biomedical applications^18^. A deeper understanding of their propulsion mechanisms, particularly in terms of magnetization, is essential for practical use. Our earlier work demonstrated that V-shaped microswimmers could be effectively propelled in Newtonian fluids^6^, with experimental results closely aligning with theoretical predictions in Morozov et al.^19^, validating rotation-based propulsion strategies in fluid environments. While fluidic propulsion is essential in many scenarios, it is not always the optimal strategy. In situations where fluid properties or flow rates make swimming less effective, surface motion becomes an important alternative. In previous studies, surface motion was controllable^18^, but a thorough analysis of its characteristics was not conducted.

The ability to alternate between fluidic and surface motion significantly enhances the versatility of these microscale robots. Surface motion is particularly useful in biomedical contexts where fluidic propulsion is less effective due to the fluid properties or high flow rates^20^. This capability is crucial for applications such as targeted drug delivery, vascular navigation, and localized diagnostics, where precise control and adaptability are required^21^. In this study, we derived the surface motion dynamics of V-shaped magnetic microswimmers, focusing on the interplay between magnetic torques, viscous drag, and frictional forces that govern stable rolling motion. By analyzing how these forces and torques interact under varying conditions, we explained the mechanisms that lead to steady rolling or unstable, wobbling behaviors.

Building on this foundation, the present study examines the surface motion of planar microswimmers under varying rotating magnetic field frequencies and field strengths. By investigating how the frequency and strength of the rotating magnetic field affect the velocity of planar microswimmers, we identified regions of both stable and unstable motion and demonstrated letter-shaped trajectory tracking. Furthermore, we showed controllable swimming motion of microswimmers within PDMS channels fabricated using 3D printed molds. The use of 3D printed molds enabled the low-cost precise design of microchannels, allowing for guidance in confined environments. Additionally, we investigated the collective behavior of microswimmer swarms under global magnetic field.

The aim of this study is to enhance the functionality of planar magnetic microswimmers, deepening the understanding of their motion across various conditions and modes. These advancements underscore the operational flexibility of the microswimmers and their potential for targeted applications in complex biomedical environments. The findings offer valuable insights that could drive the development of innovative medical treatments and diagnostic techniques.

## Fabrication and experimental setup

### Hardware setup

**Fig. 1:**
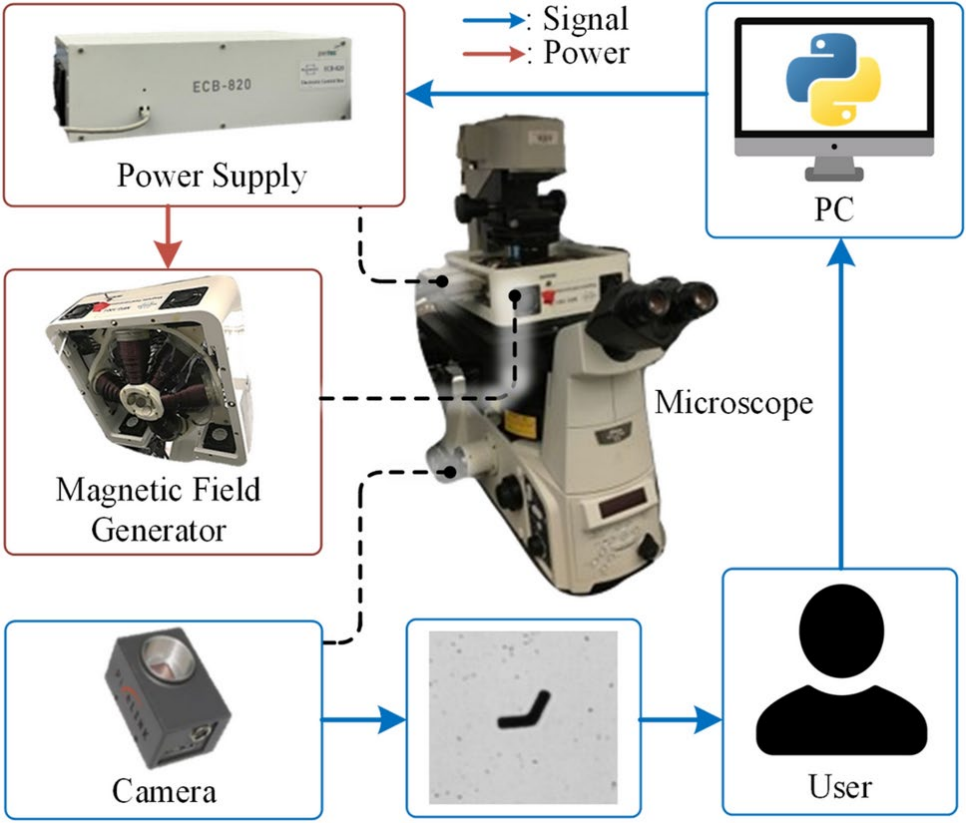
Experimental setup. For experimental purposes, the Nikon Eclipse-Ti microscope is utilized. The MagnetobiX MFG-100-I creates the magnetic field, which is controlled using MBX PRO software and an ECB-820 power supply. The Pixelink PL-D755CU-T camera is used to capture the images in real time.

Figure 1 illustrates the experimental setup. For detailed observations, a Nikon Eclipse-Ti Microscope equipped with a 10$\times$ objective lens was utilized. Conversely, to capture the entire experimental chamber, including the channels showcased in the Supporting video, a 2$\times$ objective lens was employed. Experimental videos were recorded using a high-resolution Pixelink PL-D755CU-T camera.

A MagnetobiX AG (Switzerland) MFG-100-I magnetic field generator was used to control the motion of the planar microswimmers. This generator is equipped with eight electromagnetic coils and can generate both static and rotating magnetic fields, including conical rotations, making it highly versatile for the different conditions tested in the experiments. An open-loop control approach was used, with the magnetic field parameters preset using the generator’s software interface, enabling consistent and repeatable control of the microswimmers across various field conditions.

### Fabrication of planar microswimmers

The fabrication of planar microswimmers follows conventional photolithography techniques, as detailed in Chen et al.^5^. The process begins by coating a silicon wafer with a dextran sacrificial layer, chosen for its water-soluble properties. SU-8 2005 photoresist is then applied on top of the dextran layer. Through standard photolithography steps, including soft bake, exposure, and post-exposure bake, V-shaped structures are created. To ensure the microswimmers and the subsequent nickel coating do not detach prematurely, the wafer undergoes oxygen plasma treatment to remove the dextran layer at a controlled stage. A magnetic property is imparted to the microswimmers by depositing a thin nickel layer (100 nm) over the SU-8 structures via electron-beam evaporation. The wafer, containing the V-shaped microswimmers, is then immersed in DI water, where the dextran layer dissolves. The microswimmers remain fixed to the substrate until the sacrificial dextran layer is fully dissolved.

The microswimmers in this study are crafted with a symmetric V-shaped form, optimized for propulsion by a central angle of $120^{\circ }$, as corroborated by previous theoretical research^22^. This angle is postulated to be nearly ideal for propulsion, enhancing the speed effectively. Each microswimmer measures 40 $\upmu$m in length and 20 $\upmu$m in width, with a height of 5 $\upmu$m.

### Experiment chamber and channel fabrication

**Fig. 2:**
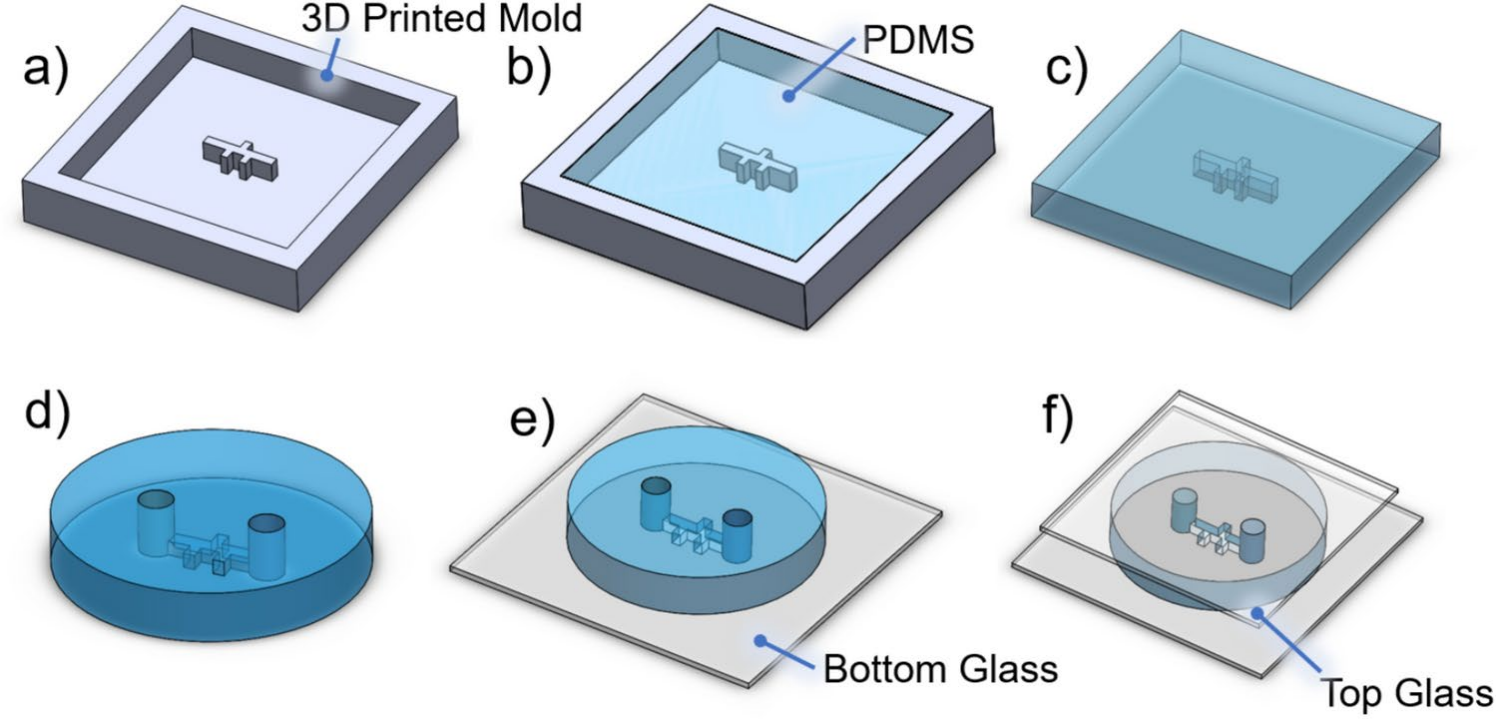
Channel fabrication procedure: (**a**) Design of the channel structure. The channel has a width of 1000 $\upmu$m and a height of 3000 $\upmu$m, facilitating microswimmer movement at a specific distance from the surface to mitigate boundary effects while displaying swimming motion. (**b**) PDMS is poured into the mold. (**c**) PDMS is cured and then removed. (**d**) Input/output ports are punched. (**e**) PDMS channel is bonded to glass. (**f**) Top glass is added post-fluid injection to prevent evaporation and contamination.

Master molds were designed using SolidWorks CAD software (Dassault Systèmes, Velizy-Villacoublay, France), with the initial model shown in Fig. 2a. The detailed mold dimensions included flow channels with a width of 1000 $\upmu$m and a height of 3000 $\upmu$m. A Photron Mono 4K SLA 3D Printer was used to fabricate these models using resin model R1515, achieving a layer thickness of 50 $\upmu$m. After printing, the molds were cleaned to remove any uncured resin and cured under UV light for 30 min.

For polydimethylsiloxane (PDMS) preparation, a 10:1 weight ratio of SYLGARD 184 Silicone Elastomer Base to SYLGARD 184 Silicone Elastomer Curing Agent was mixed, with approximately 3 grams used to fill each mold, as shown in Fig. 2b. The molds with PDMS were degassed to remove air bubbles and cured in an oven at $85\,^{\circ }\text {C}$ for 12 h. Once cured, the PDMS was carefully peeled from the mold as shown in Fig. 2c, and the inlet and outlet ports were punched using 4000 $\upmu$m diameter punches revealing the final device in Fig. 2d. This approach differs from the 3D-printed PDMS device in Duygu et al.^18^, where the input and output ports were integrated into the printed mold. By omitting the printed ports and manually punching them instead, we significantly simplified the process of removing the cured PDMS from the mold, making it much easier to handle.

Plasma treatment was then applied to both the PDMS channel and the glass substrate (No. 1, 22 mm $\times$ 30 mm) using a Harrick Plasma PDC-32G Plasma Cleaner, ensuring a strong bond to prevent any fluid leakage and maintain stable flow, as illustrated in Fig. 2e. Fluid and microswimmers were introduced into the channel through the inlet/outlet ports, and to prevent evaporation, a top glass was used, as shown in Fig. 2f.

**Fig. 3:**
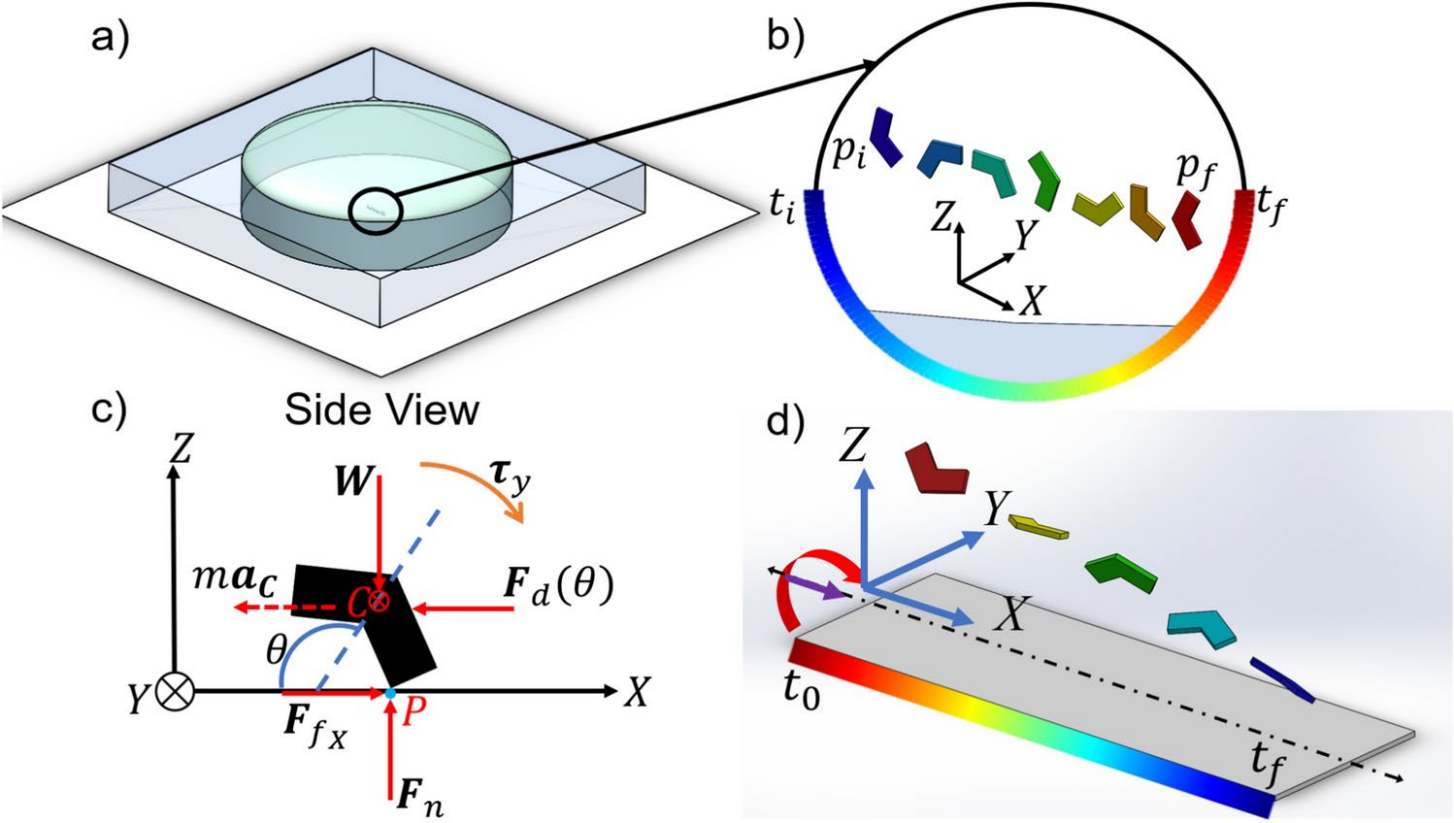
(**a**) PDMS experimental chamber used for surface motion experiments. (**b**) Visualization of the microswimmer’s surface motion, where the color gradient represents the progression of time, from the initial time $t_i$ to the final time $t_f$. The positions of the microswimmers at different points in time are shown in corresponding colors, with $p_i$ indicating the initial position and $p_f$ the final position. (**c**) Dynamics of surface rolling motion as the *X*-axis is the direction of motion where C shows the center of mass and P shows the point of contact. (**d**) Illustration of swimming motion. Reproduced with permission from IEEE^18^.

For the surface motion dynamics experiments, PDMS channels were not used, rather a PDMS chamber with a 10 mm diameter was used. PDMS chamber, filled with fluid, placed on a cover glass (No. 1, 22 mm $\times$ 30 mm) after plasma bonding similar to the channels prevent fluid leakage as depicted in Fig. 3a. Additionally, a top glass was used to reduce evaporation, dust accumulation, and internal fluid movement. The bottom of the chamber was treated with a 20% Tween20 (Sigma-Aldrich) solution to prevent microswimmers from adhering to the glass surface. Afterward, a 20% NaCl solution was introduced into the chamber, followed by the placement of planar microswimmers.

The use of a 20% NaCl solution is essential for creating a Newtonian fluid environment that aligns with our theoretical models and allows control and observation of the microswimmer’s dynamics. The salt increases the fluid’s density, which slows down the microswimmer’s sinking and makes two-dimensional control easier. This choice enhances the reliability of our experimental results and supports the validity of our conclusions regarding the microswimmer’s surface interactions and swimming motion modes.

### Microswimmer tracking

Microswimmer tracking is performed using image-based processing techniques. First, each frame of the video is converted to grayscale to simplify image data. The image is then thresholded to create a binary representation, isolating the microswimmers from the background. Contours are extracted from the binary image, and the relevant contours corresponding to microswimmers are selected based on their size and shape. By associating these contours across successive frames, we track the movement of the microswimmers.

However, challenges arise when using a 2$\times$ lens with a large region of interest (ROI), as the microswimmers appear very small. When these microswimmers are close together, the tracking becomes unreliable due to low resolution and noise. To address this, an Extended Kalman Filter (EKF) is applied to enhance tracking accuracy. The EKF predicts the microswimmer’s position in each frame and compensates for uncertainties in their motion, resulting in more reliable tracking.

This work focuses on 2D tracking of microswimmers. Future extensions could incorporate 3D tracking by using depth estimation through image blur quantification via Fast Fourier Transform (FFT), as proposed in Duygu et al.^6^, particularly for closed-loop control applications. This approach would allow for a more comprehensive analysis of microswimmer movement in three dimensions.

## Motion modes of planar microswimmers

### Surface motion

This study primarily explores surface motion, demonstrating that V-shaped planar microswimmers can achieve rolling motion. Surface motion is especially relevant in environments where swimming is constrained, such as within the narrow capillaries of the human body or in channels with rapid flows similar to those in large arterial vessels. In these settings, the restricted transverse maneuverability necessitates adopting alternative motion modes to enhance the microswimmers’ navigational capabilities.

For instance, in the confined spaces within extracellular domains or the complex networks of the lymphatic system, the ability to perform controlled surface motion is essential for effective navigation. This capability becomes crucial in navigating the dynamic and often turbulent conditions encountered within the gastrointestinal tract. Here, adapting to surface motion can enable microswimmers to maintain stability and control, allowing them to effectively handle the varying flow dynamics and physical barriers they encounter. This adaptation is vital for their potential therapeutic and diagnostic applications in medical settings, highlighting the importance of tailored motion strategies for microswimmers in biologically relevant environments.

An illustration of surface motion can be seen in Fig. 3b. A rotating magnetic field around the *Y*-axis induces a torque and rolling motion when the microswimmers are on the surface, consequently creating movement along the *X*-axis. The rotating field necessary to achieve motion in the *X*-axis can be expressed as:

1 $$\begin{aligned} \mathbf{B} = \mathbf {\hat{x}} \sin (\omega t) + \mathbf {\hat{z}} \cos (\omega t) \end{aligned}$$

where $\omega$ is the angular frequency of the field rotation, $t$ represents time, and $\mathbf {\hat{x}}$ and $\mathbf {\hat{z}}$ are the unit vectors in the *X* and *Z* directions, respectively. This equation describes a rotating magnetic field in the *XZ*-plane, changing its direction with time in a sinusoidal manner due to the cosine and sine functions.

In this study, we aim to demonstrate the dynamics of surface rolling motion, with the associated forces and torques illustrated in Fig. 3c. Our analysis focuses on a V-shaped magnetic microswimmer operating within a fluidic environment, where the motion is characterized solely by rolling. Our modeling approach differs from previous studies of spherical microswimmers^20,23,24^, which rely primarily on near-wall hydrodynamic lubrication forces. For V-shaped microswimmers, the high material density and unique geometry promote direct surface contact and reduce lubrication distance. Although this requires including contact interactions such as friction force ($\mathbf{F}_{f_x}$) which is generated by the normal force $\mathbf{F}_n$, in our model for completeness, the dominant dynamics at low Reynolds number remains driven by magnetic and drag forces. While contact effects influence stable rolling motion, they do not significantly alter the microswimmer’s velocity characteristics.

The magnitude of the drag force $\mathbf{F}_d(\theta )$, which depends on the microswimmer’s orientation $\theta$, the fluid’s dynamic viscosity $\mu$, and the velocity $\varvec{v}$, is quantitatively described by Stokes’ equation:

2 $$\begin{aligned} \mathbf{F}_d(\theta ) = \gamma (\theta ) \mu \varvec{v} \end{aligned}$$

Here, $\gamma (\theta )$ is the orientation-dependent linear drag coefficient that encapsulates the effects of the microswimmer’s geometry and its interaction with the fluid at different angles $\theta$ and $\mu$ is the dynamic viscosity. In low Reynolds number regimes, viscous forces dominate over inertial forces, resulting in the drag force being linearly proportional to the velocity $\varvec{v}$.

The dependency of $\gamma (\theta )$ arises because the geometric profile exposed to the flow changes as the robot rotates. Different orientations present varying surface areas and shapes to the fluid, altering the viscous drag experienced by the microswimmer. These variations affect the stress distribution around the swimmer, which in turn influences $\gamma (\theta )$.

Given the complex interplay between the microswimmer’s orientation and the fluid dynamics at low Reynolds numbers, analytical solutions for $\gamma (\theta )$ can be challenging to obtain. Therefore, computational fluid dynamics (CFD) simulations are essential for investigating how $\gamma (\theta )$ changes with $\theta$. CFD can provide insights into flow patterns and shear stress distributions around the swimmer, allowing for accurate determination of the drag coefficient at various orientations.

After obtaining drag force, the sum of external forces $\sum \mathbf{F}$ acting on the robot is given by:

3 $$\begin{aligned} \sum \mathbf{F} = \mathbf{W} + \mathbf{F}_{f_x} - \mathbf{F}_d(\theta ) - m \mathbf{a}_C= 0 \end{aligned}$$

where $\mathbf{W} = m\mathbf{g}$ is the weight of the robot.

The sum of moments about the center of mass $C$ incorporates the moments due to frictional and drag forces and is expressed as:

4 $$\begin{aligned} \sum \mathbf{M} = \varvec{\tau }_y + \mathbf{r}_{P/C} \times \mathbf{F}_{f_x} - \varvec{\tau }_d = \mathbf{I} \frac{d\mathbf {\varvec{\omega }}}{dt} + \varvec{\omega } \times (\mathbf{I} \varvec{\omega }) \end{aligned}$$

where $\varvec{\tau }_y$ represents magnetic torque acting on the robot, $\mathbf{r}_{P/C}$ is the position vector from the center of mass $C$ to the pivot point $P$, $\varvec{\tau }_d$ is the torque due to drag. $\frac{d\varvec{\omega }}{dt}$ is the angular acceleration ($\varvec{\alpha }$), $\mathbf{I}$ is the inertia tensor, and $\varvec{\omega }$ is the angular velocity. The term $\varvec{\omega } \times (\mathbf{I} \varvec{\omega })$ accounts for gyroscopic effects due to the rotation of the robot.

A simpler representation of the moment equation can be obtained by omitting friction and inertial contributions:

5 $$\begin{aligned} \sum \mathbf{M} = \varvec{\tau }_y - \varvec{\tau }_d = 0, \end{aligned}$$

which indicates that the drag torque ($\varvec{\tau }_d$) is entirely balanced by the magnetic torque ($\varvec{\tau }_y$). This simplified model captures the experimental observations in Sect. "Results".

The torque due to drag is further expressed as:

6 $$\begin{aligned} \varvec{\tau }_d = \sum _i \mathbf{r}_{d,i} \times \mathbf{F}_{d,i} = \zeta (\theta , \mu ) \varvec{\omega } \end{aligned}$$

where, $\mathbf{r}_{d,i}$ is the position vector from the robot’s center of mass to the point where the drag force $\mathbf{F}_{d,i}$ acts on each segment of the V-shaped structure. This vector defines the lever arm for the drag force, which is influenced by the local fluid dynamics and the specific geometry of the robot’s V-shaped surface at that position. At low Reynolds numbers, where viscous forces dominate, the drag torque $\varvec{\tau }_d$ is linearly proportional to the angular velocity $\varvec{\omega }$. This relationship is captured by the second part of the Eq. 6. Here, $\zeta (\theta , \mu )$ is the rotational drag coefficient, encapsulating the effects of the V-shaped geometry and its interaction with the fluid at different angles $\theta$ as well as the dynamic viscosity $\mu$. The linear dependence on $\varvec{\omega }$ arises from the dominance of viscous forces in the low Reynolds number regime, as described by Stokes’ Law.

### Swimming motion

Swimming motion is crucial for microswimmers because it significantly enhances functionality in biomedical applications. In fluid environments, such as the digestive system, the ability to swim enables these tiny robots to navigate through complex and dynamic spaces. Unlike surface motion, which is limited to planar or confined regions, swimming allows microswimmers to move freely in three dimensions, offering greater control over their trajectory and positioning. It is important to clarify that in this study, ’swimming’ refers to 2D propulsion in bulk fluid environments without boundary conditions and does not include motion against gravity. For details on 3D propulsion tracking, refer to our previous work^6^.

Swimming also provides a means of overcoming obstacles or bypassing areas where surface motion may be hindered, such as in vessels with varying diameters or irregular geometries.

The controlled (unidirectional) propulsion of in-plane magnetized planar microswimmers can be achieved by adding a constant magnetic field along the field rotation axis, such that the resulting field vector tracks the envelope of the cone. Cohen et al. found that within a finite range of actuation frequencies, a symmetric V-shaped planar microswimmer magnetized along its axis of symmetry exhibits unidirectional propulsion with a constant, frequency-independent velocity under a conically rotating magnetic field^25^. This theoretical result is further supported by our previous work^6^.

During the experiments, we applied a uniform, conically rotating magnetic field, as illustrated in Fig. 3d. The purple line represents the static field, while the red arrow indicates the rotating field’s direction. To initiate the movement of the microswimmers from the chamber’s bottom, we applied two orthogonal magnetic fields rotating about the *X* and *Z* axes, providing the necessary momentum to detach the microswimmers from the surface if they were adhered. To minimize boundary effects and optimize propulsion, this maneuver was followed by a conically rotating field around the *Z*-axis, propelling the microswimmers to at least 1000 $\upmu$m above the chamber floor.

A subsequent application of the conically rotating magnetic field, with a static component directed along the *Y*-axis, was then utilized to guide the microswimmers’ navigation in the *Y*-axis. However, due to gravitational force, the microswimmers experienced gradual sinking, as illustrated in Fig. 3d.

The mathematical form of the conically rotating magnetic field $\mathbf{B}$ used to propel microswimmers along the *X*-axis is given by:

$$\begin{aligned} \mathbf{B} = \mathbf {\hat{x}} \delta + \mathbf {\hat{y}} \sin (\omega t) + \mathbf {\hat{z}} \cos (\omega t) \end{aligned}$$

where $\delta$ represents the ratio of the static field to the rotating field components, $\omega$ is the angular frequency, and *t* is time. The effect of $\delta$ on the velocity is detailly investigated in Duygu et al.^6^.

### Steering strategy

To direct the swimmers along three-dimensional trajectories, such as the letter-shaped paths shown in our experiments, we employ rotations represented by the rotation matrices $\mathbf{R}_x(\theta )$, $\mathbf{R}_y(\theta )$, and $\mathbf{R}_z(\theta )$, corresponding to rotations about the *X*, *Y*, and *Z* axes, respectively. These matrices are used to rotate the Eqs. 1 and 7, enabling steering of the microswimmers in the intended direction in both surface and swimming motions. The rotation matrices are defined as follows:

8 $$\begin{aligned} \mathbf{R}_x(\theta )&= \begin{bmatrix} 1 & 0 & 0 \\ 0 & \cos (\theta ) & -\sin (\theta ) \\ 0 & \sin (\theta ) & \cos (\theta ) \end{bmatrix} \end{aligned}$$

9 $$\begin{aligned} \mathbf{R}_y(\theta )&= \begin{bmatrix} \cos (\theta ) & 0 & \sin (\theta ) \\ 0 & 1 & 0 \\ -\sin (\theta ) & 0 & \cos (\theta ) \end{bmatrix} \end{aligned}$$

10 $$\begin{aligned} \mathbf{R}_z(\theta )&= \begin{bmatrix} \cos (\theta ) & -\sin (\theta ) & 0 \\ \sin (\theta ) & \cos (\theta ) & 0 \\ 0 & 0 & 1 \end{bmatrix} \end{aligned}$$

By adjusting the field’s rotational axis, we finely control their direction and velocity along the bounding surface or bulk fluid without the need for any inverse kinematics. This approach offers new possibilities for application in complex and confined environments.

## Results

### Surface motion results

#### Motion characteristics

**Fig. 4:**
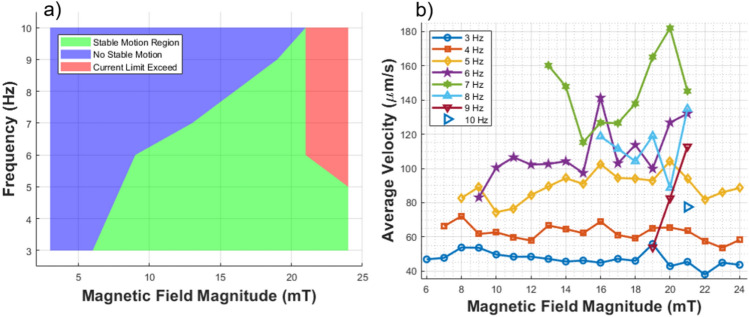
Surface motion characteristics of planar microswimmers. (**a**) The stable and unstable motion regions as a function of magnetic field magnitude and frequency. (**b**) The average surface motion velocity at different magnetic field strengths and frequencies, showing how the speed varies with these parameters.

Previous research has shown the feasibility of surface motion in planar microswimmers using a fixed frequency and magnitude of the rotating magnetic field^18^. However, that study did not examine the effects of varying magnetic field parameters on motion stability. In this study, we systematically investigate the stability of surface motion across a range of frequencies and magnitudes of the applied magnetic field, as illustrated in Fig. 4a.

This figure illustrates the regions of stable and unstable motion within the frequency-magnitude plane of the magnetic field. We evaluated the system’s performance across various magnetic field strengths and frequencies to determine whether motion in the intended direction could be achieved. Specifically, we tested magnetic field values ranging from 3 to 10 Hz and from 1 to 24 mT. The green region in Fig. 4a denotes areas of stable motion, the blue region marks areas where no stable motion was observed, and the red region indicates where the hardware’s operational limits were exceeded, preventing further exploration beyond these points.

The interplay between magnetic torque and viscous drag significantly influences the motion of magnetic microswimmers, particularly at higher frequencies. The magnetic torque, which is a crucial force enabling the microswimmers to follow the rotating magnetic field synchronously, competes with viscous drag that increases proportional the rotational speed as shown in the Eqs. 4 and  6. At high frequencies, the required synchronization leads to higher rotational speeds and, consequently, higher viscous drag. Insufficient magnetic torque, relative to the drag, causes the microswimmer to lag behind the rotating magnetic field, resulting in the wobbly or erratic motion observed. Enhancing the magnetic field strength increases the magnetic torque $\varvec{\tau }_y$, allowing the robot to better counteract the viscous drag torque $\varvec{\tau }_d$ and align more closely with the rotating field. As described in Eq. 6, the drag force experienced by the microswimmers increases linearly with the frequency of the magnetic field. To maintain synchronization at higher frequencies, the magnetic torque ($\varvec{\tau }_y$) must increase proportionally. Since the magnetic torque is directly related to the magnetic field strength, the observed near-linear relationship between step-out frequency and field strength in Fig. 4a aligns with theoretical expectations.

The stability of the microswimmer’s motion is assessed by examining its ability to follow the intended direction of the rotating magnetic field with minimal deviation. Specifically, stable motion is identified when the microswimmer moves consistently in the desired direction without excessive wiggling or erratic behavior. If wiggling becomes significant, the microswimmer tends to veer off course, often resulting in almost random movement.

This result shows that, to maintain effective control and minimize the phase lag that can occur with high-frequency rotations, it is essential to balance the magnetic torque against the viscous drag. This balancing act is critical to ensure the microswimmer performs optimal surface motion in fluid environments.

Another objective of this research is to explore how the magnitude and frequency of the rotating field influence microswimmer locomotion. Specifically, the stable surface motion of the microswimmer is illustrated in Fig. 4a, while the corresponding speed values are analyzed and presented in Fig. 4b. Each curve represents the average velocity of the microswimmer at frequencies ranging from 3 to 10 Hz, differentiated by distinct colors and symbols for clarity. The data reveal an initial increase in velocity with the magnetic field magnitude. However, for frequencies above 7 Hz, a decrease in velocity is observed, indicating completely stable motion is not achieved at these settings, possibly requiring higher field magnitudes. Within the stable regions, the magnitude of the magnetic field does not significantly affect velocity. Furthermore, increasing the frequency accentuates the peaks and troughs in velocity, highlighting that precise control of the microswimmer can be achieved by adjusting the magnetic field’s strength and frequency. For applications requiring consistent, moderate speeds, lower frequencies combined with moderate magnetic field strengths might be more useful. Lower frequencies and field magnitudes contribute to reduced energy consumption and extended experiment durations, as they prevent the coils from overheating quickly, but they also result in slower speeds.

#### Trajectory following

**Fig. 5:**
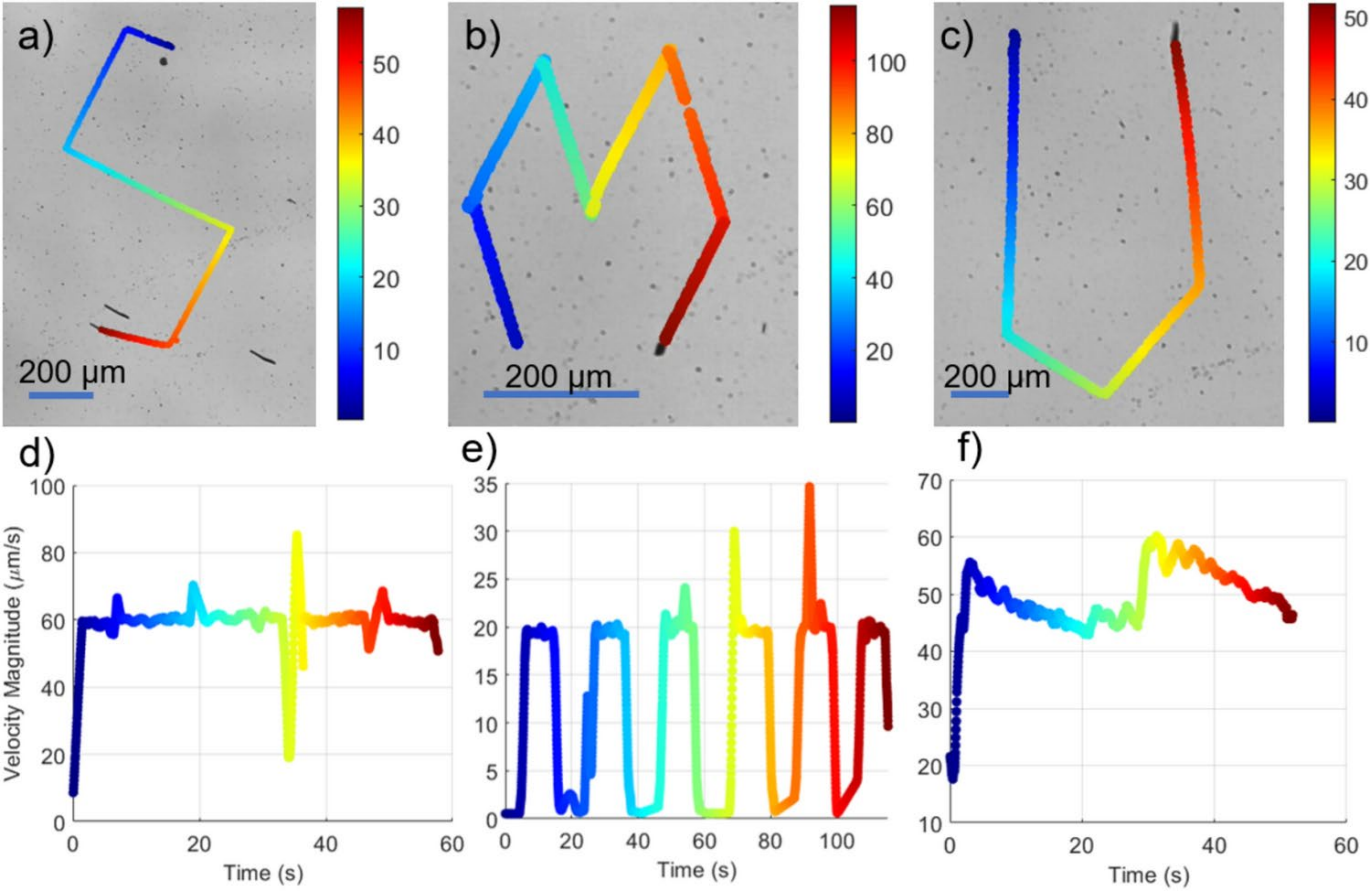
(**a**–**c**) show microswimmers tracing the paths of the letters ’S’, ’M’, and ’U’, respectively. The ’M’ shape is modeled after the distinctive diamond pattern seen in the SMU band logo. The color gradient represents the progression of time in seconds, with blue representing earlier times and red representing later times during the tracking process. This color scheme is also used in the velocity profiles in panels (**d**–**f**) to link the trajectory at a specific moment to its corresponding velocity. The blue scale bars represent 200 $\upmu$m.

The control of the microrobot is performed using an open-loop approach, where a predetermined magnetic field is applied, and the motion of the microrobot is observed without real-time feedback adjustment.

In this experimental visualization, microswimmers trace predefined paths corresponding to the letters ’S’, ’M’, and ’U’ (Fig. 5). Each sub-figure illustrates the trajectory taken by the microswimmers: ’S’ in panel (a), ’M’ in panel (b), and ’U’ in panel (c). The color gradient along the paths represents the progression of time in seconds, visually mapping the microswimmer’s movement. These experiments were conducted under a rotating magnetic field, with frequencies of 4 Hz, 2 Hz, and 3 Hz for the ’S’, ’M’, and ’U’ paths, respectively. The average velocities recorded were 60.36 $\upmu$m/s for ’S’, 19.46 $\upmu$m/s for ’M’, and 46.21 $\upmu$m/s for ’U’. Panels (d), (e), and (f) in Fig. 5 show the corresponding velocity profiles over time for each trajectory.

The large velocity fluctuations in the ’M’ trajectory occurred because the movement was paused at the edges of the ’M’ shape during the experiment. This stop-and-go movement caused the variation seen in the visualization. The data were also filtered to improve how the results are shown. For the ’S’ and ’U’ trajectories, there were no pauses during movement, so their velocity profiles appear smoother despite having corners or curves.

One such limitation of open-loop control is its inability to ensure smooth turns during complex trajectories. The sharp turns observed in the “S,” “M,” and “U” trajectories were designed this way due to the open-loop control strategy employed in this study. Open-loop control lacks the ability to adapt to real-time deviations. To achieve smoother paths, future work will focus on implementing smooth motion planning algorithms, such as polynomial trajectory generation, and combining them with a closed-loop feedback controller. This will ensure the microrobot follows the desired path accurately while maintaining gradual transitions.

The position of the microswimmers was determined using binary image processing to detect their contours in each frame. The geometric center (centroid) of the detected contour was used as the microswimmer’s position. Velocity was calculated by taking the difference in the centroid positions between successive frames and dividing by the time interval between frames. The average velocity over the trajectory was then computed by averaging these frame-to-frame velocities. Additionally, a filtering process was applied to smooth out fluctuations caused by noise, ensuring that the velocity plots accurately reflect the microswimmers’ motion dynamics.

### Swimming motion results

**Fig .6:**
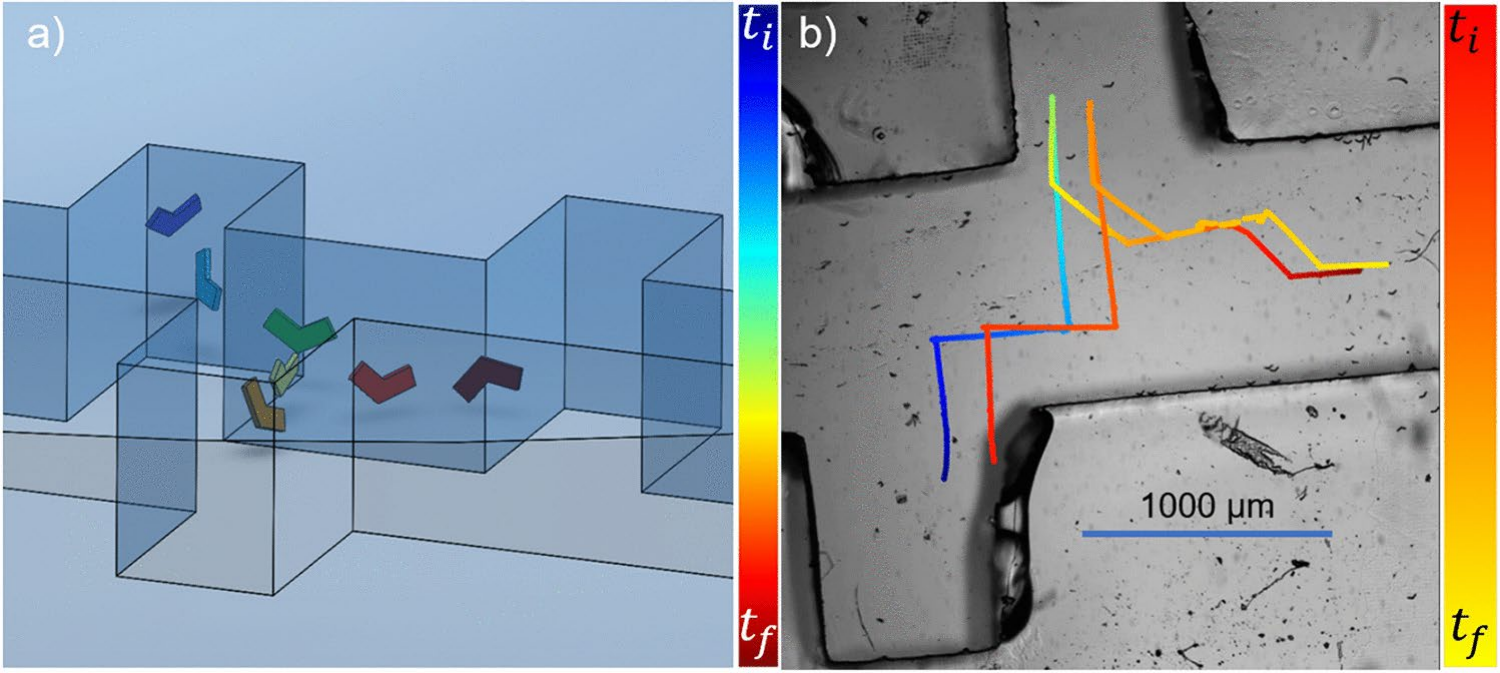
(**a**) Illustration of the microswimmer’s trajectory inside a channel (**b**) Experimental tracking of two microswimmers’ propulsion. Color gradients indicating time progression along the path, from the initial time $t_i$ to the final time $t_f$. Blue bar shows 1000 $\upmu$m.

Figure 6a illustrates a microswimmer navigating through microfabricated channels, which are extensively described in Sect. "Experiment chamber and channel fabrication". Figure 6b depicts the experimental results of the swimming motion. In these experiments, the microswimmers were guided by the steering strategy described in Sect. "Steering strategy", where direction control is achieved by varying the angle $\theta$ in the rotation matrices. In this particular experiment, only the rotation matrix $R_z(\theta )$ was used. The $\theta$ values were selected as $90^{\circ }$, $0^{\circ }$, $90^{\circ }$, $270^{\circ }$, $315^{\circ }$, $0^{\circ }$, $315^{\circ }$, and $0^{\circ }$, implementing a 2D navigation approach. Due to the higher density of the microswimmers compared to the surrounding fluid, gradual sinking was observed, which required continuous adjustment of the microscope’s focal point to accurately follow the microswimmers’ path.

The controlled propulsion of the microswimmers during swimming is frequency-independent within the operational range of the conically rotating magnetic field, as supported by theoretical and experimental findings in Cohen et al.^25^ and Duygu et al.^6^. This frequency independence differentiates swimming motion from surface motion, which is significantly influenced by the frequency and magnitude of the rotating magnetic field, as discussed in Sect. "Motion characteristics". While swimming speed data are not explicitly provided here, this independence ensures consistent performance across a wide frequency range, emphasizing the robustness of swimming motion in fluid environments.

The insights gained from these experiments underscore the need for future research to focus on the development of a 3D swimming control strategy and the integration of a closed-loop control system to enhance navigational precision and effectiveness in fluidic environments. To achieve this, rotating the axis of rotation solely around the *Z*-axis will not suffice, and the use of $R_y(\theta )$ and $R_x(\theta )$ will also be necessary. Future work will implement a 3D control strategy using the blurriness detection algorithm described in detail in Duygu et al.^6^.

The tracking results depicted in Figures 5 and 6 show that microswimmers do not always precisely follow the intended paths. This deviation can be attributed to several factors. Firstly, the use of an open-loop control strategy means that any external disturbances can alter the system’s behavior. Additionally, variations in the magnetization of the microswimmers can unintentionally change their direction of movement, further decreasing the path-following accuracy.

### Swarm applications

Swarm operation is important because it leverages the collective behavior of multiple microswimmers to achieve tasks that would be difficult or impossible for a single microswimmer. In applications such as drug delivery, where the environment is complex and unpredictable, swarms provide a built-in redundancy that ensures the overall success of the operation, even if some microswimmers fail. The distributed nature of a swarm allows for better coverage and more efficient drug distribution, as the swarm can spread out to target multiple areas simultaneously, using different magnetization directions. This collective approach may also improve navigation through obstacles. Swarms can also perform cooperative functions, such as assembling to carry larger payloads or coordinating their efforts to focus on a specific target, making them versatile and resilient. Moreover, the scalability of swarm systems allows them to be fine-tuned for different medical applications, from localized drug delivery to more extensive treatments, enhancing both precision and efficacy.

**Fig .7:**
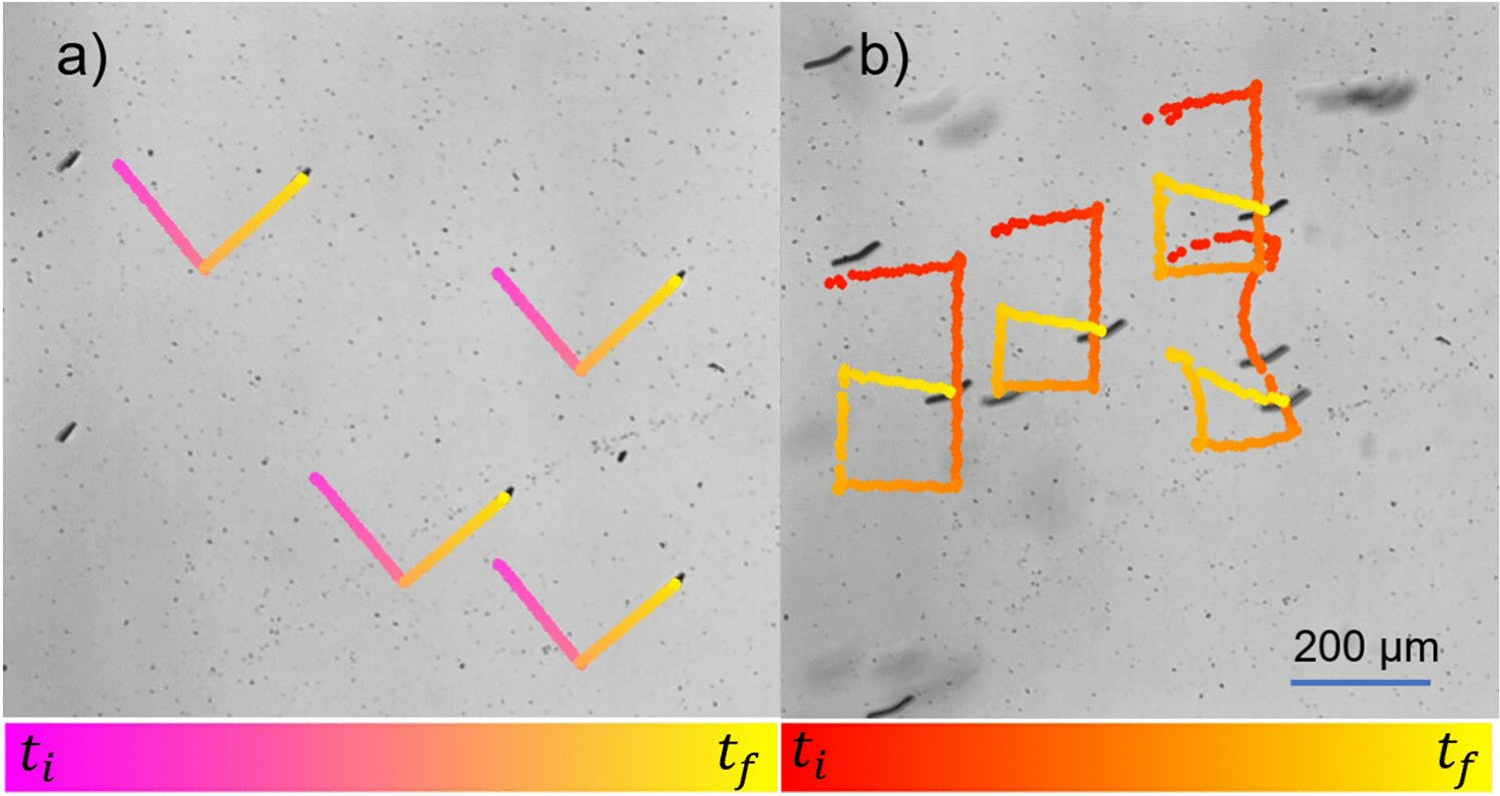
(**a**) Experimental tracking of “V” shaped trajectory of surface motion. (**b**) Experimental tracking of the microswimmer propulsion following “a” shaped trajectories of microswimmers. In both cases, swarm control of microswimmers is achieved using a global magnetic field. Color gradients indicate time progression along the path, from the initial time $t_i$ to the final time $t_f$. Figures (**a**) and (**b**) are reproduced with permission from IEEE^18^.

#### Surface swarm motion

As described in Sect. "Steering strategy", by adjusting the field’s rotational axis, we can precisely control the direction and velocity of the microswimmers along the surface. Figure 7a illustrates the controlled surface movement of the planar microswimmers, accurately following the outlined paths for the letter “V,” which represents the V-shaped geometry of our microswimmer. The experiments were conducted with a 5 mT in-plane rotating magnetic field at a frequency of 1 Hz. The average velocity recorded was 11.72 $\upmu$m/s, which is considerably lower than the velocities observed at higher frequencies, as discussed in Sect. "Motion characteristics".

#### Swimming swarm motion

Experiments were also conducted to evaluate the group swimming behavior of microswimmers, as illustrated in Fig. 7b, where multiple units are observed following an ’a’-shaped trajectory in unison. However, variations in trajectory paths and velocities were noted among the microswimmers. These discrepancies in velocity may be attributed to slight differences in the magnetization orientation and strength of individual microswimmers, likely caused by manufacturing inconsistencies. The deviations in trajectory, particularly seen in the microswimmer at the lower right side of Fig. 7b, drifting from the planned path, could result from hydrodynamic interactions between the microswimmers.

Each spinning propeller generates a localized swirling flow, effectively acting as a point rotlet, creating a vortex-like flow around it^26^. While this swirling flow does not significantly affect the collective movement of the microswimmers, it can influence their individual paths. The impact of this flow decreases with the square of the distance from the source, so the behavior of nearby microswimmers is largely unaffected when they maintain a separation distance, $l$, that exceeds the size of an individual propeller^26^. Initially positioned at this separation distance $l$, the microswimmers experience hydrodynamic interactions, which contribute to the altered trajectories observed. As shown in Fig. 7a, the deviation from the intended path is significantly lower during surface motion. This is because the boundary effects provide stability by counteracting hydrodynamic forces or disturbances, such as fluid flows in the experimental chamber.

## Conclusion

This study has significantly enhanced the understanding and operational capabilities of planar magnetic microswimmers by investigating their dual motion modes-surface rolling and fluidic swimming-and their responsiveness to varying magnetic field parameters. Through precise fabrication using standard photolithography and the application of a versatile magnetic field generator, we demonstrated the ability of V-shaped microswimmers to seamlessly transition between surface and swimming motions. Our experiments highlighted the critical balance between magnetic torque and viscous drag, identifying stable and unstable motion regions based on the frequency and magnitude of the rotating magnetic field. The successful tracing of predefined letter-shaped trajectories and navigation through 3D printed PDMS channels underscores the potential of these microswimmers for targeted biomedical applications such as drug delivery and precision surgery. Additionally, we demonstrated the collective dynamics of microswimmer swarms under a global magnetic field, suggesting new possibilities for coordinated control in larger-scale operations, where swarm behavior provides redundancy and can improve overall success rates.

However, deviations in trajectory following highlighted limitations of the current open-loop control strategy, mainly due to external disturbances and variations in microswimmer magnetization. To overcome these challenges, future work should include three-dimensional swimming and surface navigation techniques and integrate closed-loop feedback systems to enhance control precision and reliability. By advancing control mechanisms and expanding the operational flexibility of planar magnetic microswimmers, this research paves the way for their effective deployment in complex and dynamic biological environments, ultimately contributing to innovative solutions in medical diagnostics and therapeutics.

## Supplementary Information

Supplementary Information 1.

Supplementary Information 2.

## Data Availability

The data that support the findings of this study are available from the corresponding author upon request. The experimental video related to the figures is provided as Supplementary Video 1.
