# Supplementary material for: Surface motion dynamics and swimming control of planar magnetic microswimmers
Source: Sci Rep. 2025 Mar 20;15:9645. doi: 10.1038/s41598-025-94078-y (PMC11926103; doi:10.1038/s41598-025-94078-y)
Supplement: Supplementary file 1 — Supplementary Information 1. [file 41598_2025_94078_MOESM1_ESM.pdf]

Legend for Supplementary Video:

This video presents the experimental results corresponding to Figures 5(a), 5(b), 5(c), 6(b), 7(a), and 7(b). It visually demonstrates the microswimmer's response under the described experimental conditions.
